# Early-Phase Recovery of Cardiorespiratory Measurements after Maximal Cardiopulmonary Exercise Testing in Patients with Chronic Obstructive Pulmonary Disease

**DOI:** 10.1155/2016/9160781

**Published:** 2016-11-27

**Authors:** Marie Bellefleur, David Debeaumont, Alain Boutry, Marie Netchitailo, Antoine Cuvelier, Jean-François Muir, Catherine Tardif, Jérémy Coquart

**Affiliations:** ^1^Service de Pneumologie, Hôpital de Bois-Guillaume, CHU de Rouen, 76031 Rouen Cedex, France; ^2^Service de Physiologie Digestive, Urinaire, Respiratoire et Sportive, CHU de Rouen, 76000 Rouen, France; ^3^UPRES EA 3830, GRHV, 76000 Rouen, France; ^4^CETAPS, EA 3832, UFR STAPS, Université de Rouen, 76821 Mont Saint Aignan, France

## Abstract

*Background*. This study investigated respiratory gas exchanges and heart rate (HR) kinetics during early-phase recovery after a maximal cardiopulmonary exercise test (CPET) in patients with chronic obstructive pulmonary disease (COPD) grouped according to airflow limitation.* Methods*. Thirty control individuals (control group: CG) and 81 COPD patients (45 with “mild” or “moderate” airflow limitation, COPD_I-II_, versus 36 with “severe” or “very severe” COPD, COPD_III-IV_) performed a maximal CPET. The first 3 min of recovery kinetics was investigated for oxygen uptake ($\dot{V}$O_2_), minute ventilation (${\dot{V}}_{E}$), respiratory equivalence, and HR. The time for $\dot{V}$O_2_ to reach 25% (T_1/4_ $\dot{V}$O_2_) of peak value was also determined and compared.* Results*. The $\dot{V}$O_2_, ${\dot{V}}_{E}$, and HR recovery kinetics were significantly slower in both COPD groups than CG (*p* < 0.05). Moreover, COPD_III-IV_ group had significantly higher $\dot{V}$O_2_ and ${\dot{V}}_{E}$ during recovery than COPD_I-II_ group (*p* < 0.05). T_1/4_ $\dot{V}$O_2_ significantly differed between groups (*p* < 0.01; 58 ± 18 s in CG, 79 ± 26 s in COPD_I-II_ group, and 121 ± 34 s in COPD_III-IV_) and was significantly correlated with forced expiratory volume in one second in COPD patients (*p* < 0.001, *r* = 0.53) and with peak power output (*p* < 0.001, *r* = 0.59).* Conclusion*. The COPD groups showed slower kinetics in the early recovery period than CG, and the kinetics varied with severity of airflow obstruction.

## 1. Introduction

Patients with chronic obstructive pulmonary disease (COPD) are characterized by dyspnea and physical exercise intolerance, both of which impair the ability to participate in physical activities and lower the quality of life [1–3]. The cardiopulmonary exercise test (CPET) is the standard test to investigate the pathophysiological mechanisms of dyspnea and evaluate physical exercise tolerance [4, 5]. Indeed, exercise intolerance in COPD patients is often demonstrated during CPET by an early ventilatory threshold and/or low peak oxygen uptake ($\dot{V}$O_2_peak). However, the cardiorespiratory data measured during the recovery period after CPET may also provide indications of physical fitness.

Oxygen uptake ($\dot{V}$O_2_) and heart rate (HR) kinetics during recovery after physical exercise are indicators of physical fitness and cardiovascular health [6]. The recovery period has been widely studied in healthy individuals and patients with cardiac disease [7–9]. For example, the $\dot{V}$O_2_ kinetics during recovery after submaximal and maximal exercise is prolonged in heart failure patients and closely correlated with indicators of physical fitness (*e.g.*, $\dot{V}$O_2_peak). Thus, authors attempted to find physical fitness indicators from the physiological data collected during the recovery period [7].

The half-time recovery of $\dot{V}$O_2_ (*i.e.*, T_1/2_ $\dot{V}$O_2_), which is the time required for a 50% fall in the $\dot{V}$O_2_peak, was identified as an indicator of physical fitness in patients with cardiac disease [7]. However, few studies have examined T_1/2_ $\dot{V}$O_2_ or other potential indicators of physical fitness (such as T_1/4_ $\dot{V}$O_2_, which corresponds to quarter-time of recovery in $\dot{V}$O_2_) in patients with respiratory diseases, especially COPD [10].

The aim of the current study was to examine the respiratory gas exchanges (specifically for $\dot{V}$O_2_) and HR kinetics during the early recovery phase after a maximal CPET according to COPD severity. We hypothesized that $\dot{V}$O_2_ and HR kinetics would be significantly slower in patients with severe COPD.

## 2. Materials and Methods

### 2.1. Population

In this retrospective, observational, routine clinical practice study, all control individuals and COPD patients (men and women) who had performed a CPET on a cycle ergometer between January 1st 2012 and December 31st 2015 at the Rouen University Hospital, France, were included. All procedures performed in this study were in accordance with the ethical standards of the institutional and national research committee and with the 1964 Helsinki declaration and its later amendments.

Thirty control individuals (*i.e.*, control group: CG) and 81 COPD patients participated in the study (Table 1). The COPD patients were assigned to one of two groups depending on the severity of airflow limitation (*i.e.*, COPD_I-II_, 45 patients with “mild” or “moderate” airflow limitation, versus COPD_III-IV_, 36 patients with “severe” or “very severe” COPD; Table 1).

Exclusion criteria were respiratory diseases other than COPD, recent exacerbation (in the 6 months preceding inclusion), chronic heart failure (left ventricular ejection fraction < 55%), undernutrition (body mass index: BMI < 18.5 kg·m^−2^), severe obesity (BMI ≥ 35 kg·m^−2^), and any muscular or metabolic disease. Medications that could influence heart rate and arterial tension are presented in Table 2. Moreover, when the physician decided to stop the CPET because of myocardial ischemia, arrhythmia, or systemic hypertension, the participant was excluded. Last, none of the control individuals or patients was engaged in an exercise training program prior to the study, and none of the participants was considered as very deconditioned (*i.e.*, ventilatory threshold < 40%  $\dot{V}$O_2_peak).

### 2.2. Protocol

Before CPET, anthropometric (*i.e.*, height and BMI) and spirometric (*i.e.*, forced expiratory volume in one second (FEV_1_) and forced vital capacity (FVC)) data were collected for each participant. The percentage of the vital capacity expiring in the first second of maximal expiration (*i.e.*, FEV_1_/FVC × 100) was calculated in order to confirm COPD (FEV_1_/FVC × 100 < 70%). The severity of airflow limitation was determined from FEV_1_, according to the Global Initiative for Chronic Obstructive Lung Disease [11]. When FEV_1_/FVC × 100 ≥ 70%, the patient was included in the control group.

Then, participants performed a symptom-limited CPET on an electromagnetically braked cycle ergometer (BV Lode®, Groningen, Netherlands) in accordance with Palange et al. [12]. Following a 3-minute warm-up, power output gradually increased every minute with increments of 5 to 20 W according to the patient's fitness level and severity of airflow obstruction until the point of maximal effort (*i.e.*, the participant failed to maintain a pedaling rate above 60 rpm for more than 5 s, unless the test was terminated for medical reasons). The initial and subsequent increments in power output were set to ensure exercise duration between 8 and 12 min. A pedaling rate of 60–70 rpm was maintained throughout CPET. The patients were instructed to attain the highest possible power output. After the point of maximal effort, a 3-minute recovery period was investigated. Expired air (*i.e.*, $\dot{V}$O_2_, carbon dioxide output: $\dot{V}$CO_2_, and minute ventilation: ${\dot{V}}_{E}$) was continuously recorded via a breath-by-breath system (Medisoft®, Sorinnes, Belgium), calibrated in accordance with the manufacturer's guidelines (before each CPET) and averaged every 1 min (over 30 s). HR was also recorded with a 12-lead electrocardiogram (Medcard, Medisoft®, Sorinnes, Belgium).

Maximal effort was checked according to the following criteria: (1) respiratory exchange ratio ≥ 1.1, (2) ventilatory reserve ≤ 30%, (3) peak HR ≥ 90% of the theoretical peak HR (*i.e.*, 210 − 0.65 × age), and (4) subjective state of extreme physical tiredness. In all cases, at least three of the four criteria were met, or the participant was excluded. When maximal effort was confirmed, actual $\dot{V}$O_2_peak was considered as the highest $\dot{V}$O_2_ over 30 s. At this point, respiratory gas exchanges and HR were collected.

During the recovery period, $\dot{V}$O_2_, $\dot{V}$CO_2_, ${\dot{V}}_{E}$, and HR were measured for 3 min and averaged every 1 min (over 30 s). Oxygen equivalence (EqO_2_ = ${\dot{V}}_{E}$/$\dot{V}$O_2_) and carbon dioxide equivalence (EqCO_2_ = ${\dot{V}}_{E}$/$\dot{V}$CO_2_) were computed, and the time needed for $\dot{V}$O_2_ to reach 25% of its peak value was recorded (T_1/4_ $\dot{V}$O_2_).

### 2.3. Statistical Analysis

Data are reported as means and standard deviation. For all data, normal Gaussian distributions were verified by the Shapiro-Wilk test and homogeneity of variance by the Levene test. When the data did not pass the test for normality and/or homogeneity of variance, they were log transformed.

For anthropometric and spirometric data, a general linear model with a 1-way design was used to test the group effect (*i.e.*, CG versus COPD_I-II_ versus COPD_III-IV_). If significant differences were obtained, a Bonferroni* post hoc* test was conducted.

Possible differences in the recovery period were tested using the general linear model (groups) for repeated measures (*i.e.*, recovery period: 0, 60, 120, and 180 s). The sphericity was checked by the Mauchly test and, when it was not met, the significance of* F*-ratios was adjusted according to the Greenhouse-Geisser procedure or the Huynh-Feldt procedure. When significant differences were obtained, a Bonferroni* post hoc* test was conducted.

For T_1/4_ $\dot{V}$O_2_, a general linear model with a 1-way design was used to examine the group effect (*i.e.*, CG versus COPD_I-II_ versus COPD_III-IV_). If significant differences were obtained, a Bonferroni* post hoc* test was conducted.

The Pearson product moment correlation was used to evaluate the association between T_1/4_ $\dot{V}$O_2_ and peak power output and FEV_1_ in COPD groups.

Statistical significance was set at *p* < 0.05 and all analyses were performed with the Statistical Package for the Social Sciences (release 18.0, Chicago, IL, USA).

## 3. Results

Anthropometric and spirometric data are summarized in Table 1.

Patients in CG were significantly younger than those in the COPD groups (*p* < 0.001, Table 1). BMI was significantly lower in COPD_III-IV_ compared with COPD_I-II_ (*p* < 0.001).

Logically, FEV_1_ and FVC (both expressed in L and %) were significantly different between groups (*p* < 0.001, Table 1). Inspiratory capacity/total lung capacity and diffusing capacity of the lungs for carbon monoxide were significantly higher in CG compared with the COPD groups, and these values were significantly lower in COPD_III-IV_ compared with COPD_I-II_ (*p* < 0.05, Table 1).

At maximal effort, $\dot{V}$O_2_peak and peak of ${\dot{V}}_{E}$ were significantly higher in CG compared with the COPD groups (*p* < 0.001, Table 1). Moreover, these values were significantly lower in COPD_III-IV_ than COPD_I-II_ (*p* < 0.001). For peak of HR, the only significant difference was noted between CG and the two COPD groups (*p* < 0.001, Table 1).

**Figure 1:**
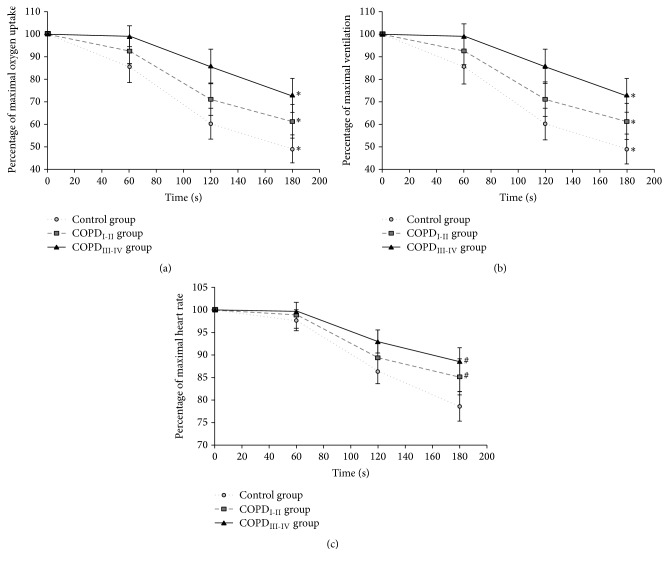
Kinetics of recovery of oxygen consumption (a), ventilation (b), and heart rate (c) according to groups. COPD: chronic obstructive pulmonary disease. ^**∗**^Significantly different from both groups (*p* < 0.05). ^#^Significantly different from control group (*p* < 0.05).

After peak exercise, all physiological data (*i.e.*, $\dot{V}$O_2_, ${\dot{V}}_{E}$, EqO_2_, EqCO_2_, and HR) were significantly different at each minute of recovery (*p* < 0.05), except HR between the peak exercise and 1st min of recovery (*p* = 0.07) and EqO_2_ between the 2nd and 3rd minutes (*p* = 0.06), while nonsignificant trends were noticed.  Figure 1 shows that the recovery kinetics of $\dot{V}$O_2_, ${\dot{V}}_{E}$, and HR were significantly slower in both COPD groups than CG (*p* < 0.05, Figure 1). Moreover, COPD_III-IV_ group had significantly higher $\dot{V}$O_2_ and ${\dot{V}}_{E}$ during the recovery in comparison with COPD_I-II_ group (*p* < 0.05).

HR decreased by 12, 7, and 5 bpm each minute during the recovery period, respectively, in CG, COPD_I-II_, and COPD_III-IV_. Moreover, HR at the first minute was decreased by 5, 3, and 1 bpm, respectively, in CG, COPD_I-II_, and COPD_III-IV_.

**Figure 2:**
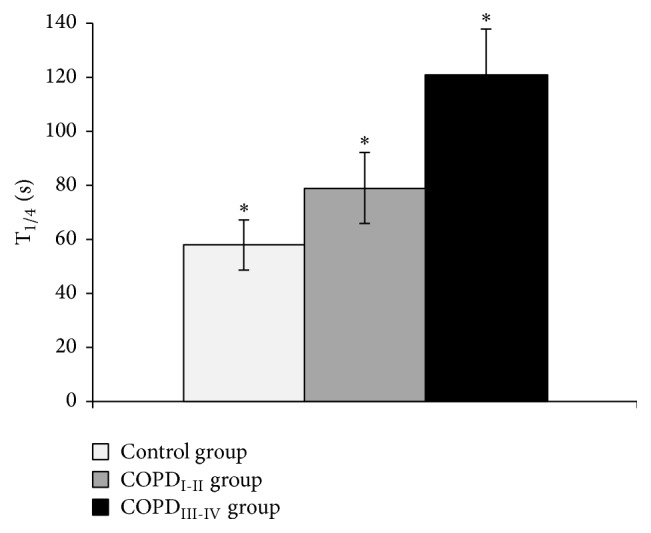
Bar graph of the mean values of quarter-time of recovery in oxygen uptake (T_1/4_) according to groups. COPD: chronic obstructive pulmonary disease. ^**∗**^significantly different from both groups (*p* < 0.01).

Figure 2 shows a bar graph of the mean values of T_1/4_ $\dot{V}$O_2_ in the three groups. T_1/4_ $\dot{V}$O_2_ was 58 ± 18 s in CG, 79 ± 26 s in COPD_I-II_, and 121 ± 34 s in COPD_III-IV_. Significant differences between groups were observed (*p* < 0.01). Moreover, T_1/4_ $\dot{V}$O_2_ was significantly correlated with FEV_1_ in the COPD patients (*p* < 0.001, *r* = 0.53) and with peak power output (*p* < 0.001, *r* = 0.59).

## 4. Discussion

The aim of the current study was to examine the $\dot{V}$O_2_, ${\dot{V}}_{E}$, EqO_2_, EqCO_2_, and HR kinetics during the early recovery phase after a maximal CPET, according to the COPD severity. Our main results showed slower recovery kinetics in both COPD groups compared with CG, with significantly longer T_1/4_ $\dot{V}$O_2_ in the COPD groups that varied with the severity of airflow obstruction.

After an (acute) aerobic exercise, $\dot{V}$O_2_ does not immediately return to the resting value. This “excess postexercise oxygen consumption” (EPOC) [13] has an immediate phase during which oxygen is required to rebuild adenosine triphosphate and phosphocreatine. Then, in the following minutes, EPOC is thought to be implicated in the removal of accumulated lactate acid [13]. The recovery period is thus devoted to the repayment of the oxygen debt generated during physical exercise. The high EPOC noted in the current study (Figure 1) may be partially due to the muscle dysfunctions common to COPD patients. Indeed, these patients often show a decreased capacity for aerobic energy metabolism manifested by an increased recovery time for phosphocreatine, as previously shown by 31-phosphorous magnetic resonance spectroscopy [14, 15].

Prolonged $\dot{V}$O_2_ kinetics during the recovery period have been observed in various situations like deconditioning [16] and COPD [10]. For example, all respiratory gas exchanges were slowed in COPD patients compared with healthy individuals [10]. The present results confirm these findings, with a significantly slower recovery of $\dot{V}$O_2_ and ${\dot{V}}_{E}$ (Figure 1). The physiological mechanisms underlying the slower kinetics during exercise recovery are not completely understood, but part of the explanation is the slow recovery of energy stores in peripheral skeletal muscles, as proposed by Thompson et al. [17].

The insidious development of airflow limitation and dynamic hyperinflation over many years in COPD patients leads to some structural and mechanical adaptations, which preserve the functional strength of the overburdened inspiratory muscles, particularly the diaphragm [18, 19]. It is thought that the function of intercostal and sternomastoid muscles is at less of a disadvantage compared to the diaphragm in the presence of severe hyperinflation [20]. However, despite this temporal adaptation, the presence of severe hyperinflation means that the ability to increase ventilation when the demand arises is greatly limited in COPD patients [18]. Consequently, this constraint may probably explain the slower recovery of ${\dot{V}}_{E}$ reported in our patients with severe COPD.

Cohen-Solal et al. [7] reported that T_1/2_ $\dot{V}$O_2_ was an indicator of physical fitness in patients with chronic heart failure. However, T_1/2_ $\dot{V}$O_2_ determination requires a long recovery period (often 6 min or more). For example, in the current study, it was not possible to determine T_1/2_ $\dot{V}$O_2_ for 9 individuals in CG, 25 patients in COPD_I-II_, and 25 patients in COPD_III-IV_. We therefore preferred to use T_1/4_ $\dot{V}$O_2_. This indicator is interesting primarily because it is a potential alternative to T_1/2_ $\dot{V}$O_2_. Indeed, T_1/4_ $\dot{V}$O_2_ was significantly different between groups (*i.e.*, higher in COPD patients) and significantly linked with disease severity (*i.e.*, FEV_1_). Therefore, if the validity, reliability, sensitivity, and usefulness of T_1/4_ $\dot{V}$O_2_ are confirmed in COPD patients, this indicator might be used as a criterion for therapeutic interventions that decrease operating lung volumes with oxygen supplementation or acute bronchodilator therapy during the recovery period [21–23]. Further studies are nevertheless needed before T_1/4_ $\dot{V}$O_2_ can be considered for routine clinical practice.

Continuous aerobic exercise has beneficial effects on patients with severe COPD [24, 25]. However, interval training, which consists of regularly alternating high intensity exercise and low intensity recovery periods, seems to provide better clinical and physiological benefits to patients with moderate and severe COPD, including increased total exercise duration, application of intense loads on peripheral muscles for sufficiently long periods of time, lower minute ventilation, lower rates of dynamic hyperinflation, and enhanced exercise tolerance and quality of life [26–28]. Therefore, interval training may be better than continuous exercise for COPD patients. The interval training protocols are nevertheless heterogeneous and no consensus exists about the optimal intensity and duration of the two phases (*i.e.*, effort and recovery periods), although these exercise characteristics (*i.e.*, intensity and duration) are major determinants of the physiological adaptations that occur during training programs [29]. The present study suggests that the duration of each phase must be individualized on the basis of the COPD severity, with a longer recovery period for patients with severe COPD. Further studies are required to investigate the appropriate time and/or interval training workload for these patients.

As indicated in the Methods section, the COPD patients were assigned to one of two groups based on the severity of airflow limitation (*i.e.*, COPD_I-II_ versus COPD_III-IV_). It might have been preferable to classify the patients into four groups to examine the sensitivity of T_1/4_ $\dot{V}$O_2_ in greater detail, but our population included only ten patients with mild COPD (*i.e.*, COPD_I_) and six patients with very severe COPD (*i.e.*, COPD_IV_). It thus was not possible to perform statistical analyses on four groups of COPD patients because of the small sample sizes.

Another possible limitation of the current study concerns the selected exhaustion criteria. Indeed, although the maximal effort was confirmed from at least three of the four exhaustion criteria, it is not sure that all participants have achieved their maximal capacity. For example, a ventilatory reserve ≤ 30% was used to check the ventilatory limitation. This threshold (*i.e.*, value ≤ 30%) is commonly used in the literature [4, 30, 31], but it remains wide, and it may not be adapted for all individuals. Indeed, recently Mirdamadi et al. [32] have reported a mean ventilatory reserve = 30.9% (which is according to our study), but their study revealed also wide standard deviation (±25.1%). More specifically, their results showed a ventilatory reserve = 43.1 ± 25.6% in patients with leg fatigue and only a ventilatory reserve = 16.6 ± 15.4 in patients without leg fatigue [32]. Consequently, it is possible that in the current study we have considered that CPET was maximal from ventilatory reserve ≤ 30% in COPD patients without leg fatigue, while a low ventilatory reserve should have been researched (approximately ≤ 15% in these patients).

Moreover, as recently reminded by Ha et al. [33], numerous authors have found a large variability in the decrease of HR at 1 min following cessation of exercise (decrease of 8–17 beats when the HR recovery is abnormal), though abnormal HR recovery is commonly defined as ≤ 12–14 [34, 35]. Consequently, the results of the present study are surprising because we noticed only a mean decrease of 5 beats in CG. This result may be explained by the possible submaximal CPET (as explained before) and/or the drugs consumed by the participants. Indeed, numerous individuals (in CG and COPD groups) consumed drugs that could influence HR and arterial tension (Table 2). These drugs have limited HRmax and thus the reduction of HR at the first minute of recovery period. Furthermore, the abnormal delayed decrease of HR in COPD and control groups may be also explained by important deconditioning in both populations.

## 5. Conclusion

The current study showed slower kinetics in the early recovery period in both COPD groups compared with CG, according to the severity of airflow obstruction. Moreover, T_1/4_ $\dot{V}$O_2_ also increased with the severity of COPD. These results may be explained by the disease and might be taken into account in the prescription of interval training exercises.

## Figures and Tables

**Table 1:** Mean ± standard deviation of anthropometric and spirometric data and physiological data measured at voluntary exhaustion according to groups.

Variables	Control group (*n* = 30)	GOLD_I-II_ group (*n* = 45)	GOLD_III-IV_ group (*n* = 36)	All participants (*n* = 111)
Age (y)	50.2 ± 15.0	60.1 ± 9.0^a^	60.8 ± 10.2^a^	57.6 ± 12.1
BMI (kg·m^−2^)	24.9 ± 4.3	27.7 ± 5.2^b^	23.4 ± 4.1	25.6 ± 5.0
FEV_1_ (L)	3.242 ± 0.886^b^	2.062 ± 0.616^a,b^	1.094 ± 0.298^a^	2.067 ± 1.036
FEV_1_ (%)	105.6 ± 12.9^b^	71.3 ± 12.3^a,b^	39.2 ± 8.2^a^	70.2 ± 27.9
FVC (L)	4.126 ± 1.160^b^	3.526 ± 0.868^a,b^	2.805 ± 0.740^a^	3.454 ± 1.045
FVC (%)	109.8 ± 14.6^b^	96.0 ± 13.4^a,b^	77.8 ± 13.1^a^	93.8 ± 18.4
FEV_1_/FVC (%)	78.8 ± 5.8^b^	59.2 ± 8.8^a,b^	41.3 ± 8.3^a^	58.7 ± 16.5
IC/TLC (%)	45.6 ± 5.9^b^	38.4 ± 8.9^a,b^	28.3 ± 6.3^a^	35.2 ± 9.7
DLCO (mmol·kPa^−1^·min^−1^)	6.737 ± 1.420^b^	5.351 ± 2.240^a,b^	3.632 ± 1.821^a^	5.035 ± 2.244
$\dot{V}$O_2_peak (L·min^−1^)	2.049 ± 0.876^b^	1.410 ± 0.508^a,b^	0.933 ± 0.232^a^	1.428 ± 0.713
${\dot{V}}_{E}$peak (L·min^−1^)	82.8 ± 35.2^b^	66.5 ± 21.7^a,b^	40.4 ± 9.9^a^	62.4 ± 28.7
HRpeak (bpm)	164 ± 17	136 ± 20^a^	127 ± 19^a^	140 ± 24

COPD: chronic obstructive pulmonary disease; *n*: sample size; BMI: body mass index; FEV_1_: forced expiratory volume in 1 second; FVC: forced vital capacity; IC: inspiratory capacity; TLC: total lung capacity; DLCO: diffusing capacity of the lungs for carbon monoxide; $\dot{V}$O_2_peak: peak oxygen uptake; ${\dot{V}}_{E}$peak: peak minute ventilation; HRpeak: peak heart rate. ^a^Significantly different from control group (*p* < 0.05). ^b^Significantly different from COPD_III-IV_ group (*p* < 0.05).

**Table 2:** Medication that could influence heart rate and arterial tension.

Variables	Control group (*n* = 30)	COPD group (*n* = 81)
Short-acting *β*_2_-agonists (*n*)	0	69
Long-acting *β*_2_-agonists (*n*)	0	48
Ipratropium bromide (*n*)	0	19
Theophylline (*n*)	1	0
Calcium channel blockers (*n*)	6	17
Angiotensin converting enzyme inhibitors (*n*)	3	11
Angiotensin II receptor antagonists (*n*)	3	10
Leukotriene receptor antagonist (*n*)	1	3
Diuretics (*n*)	4	8

COPD: chronic obstructive pulmonary disease; *n*: sample size.

## Abbreviations

BMI: Body mass index
CG: Control group
COPD: Chronic obstructive pulmonary disease
COPD_I-II_: Group with “mild” or “moderate” airflow limitation
COPD_III-IV_: Group with “severe” or “very severe” airflow limitation
CPET: Cardiopulmonary exercise test
EPOC: Excess postexercise oxygen consumption
EqCO_2_: Carbon dioxide equivalence
EqO_2_: Oxygen equivalence
FEV_1_: Forced expiratory volume in one second
FVC: Forced vital capacity
HR: Heart rate
$T_{1/2}\dot{V}O_{2}$: Half-time recovery of oxygen uptake
$T_{1/4}\dot{V}O_{2}$: Quarter-time recovery of oxygen uptake
${\dot{V}}_{E}$: Minute ventilation
$\dot{V}{CO}_{2}$: Carbon dioxide output
$\dot{V}O_{2}$: Oxygen uptake
$\dot{V}O_{2}$peak: Peak oxygen uptake.
